# Intensivbettenbedarf für COVID‑19 im Herbst/Winter 2021: Simulation unterschiedlicher Szenarien unter Berücksichtigung von Inzidenzen und Impfquoten

**DOI:** 10.1007/s00063-021-00862-9

**Published:** 2021-08-09

**Authors:** Andreas Schuppert, Steffen Weber-Carstens, Christian Karagiannidis

**Affiliations:** 1 Institute for Computational Biomedicine, Universitätsklinikum Aachen, RWTH Aachen, Pauwelsstraße 19, 52074 Aachen, Deutschland; 2 Klinik für Anästhesiologie und operative Intensivmedizin (CCM, CVK), Charité – Universitätsmedizin Berlin, Berlin, Deutschland; 3 ARDS- und ECMO-Zentrum Köln-Merheim, Kliniken der Stadt Köln, Universität Witten/Herdecke, Ostmerheimer Str. 200, 51109 Köln, Deutschland

**Keywords:** ARDS, ICU, Modell, Szenario, SARS-CoV-2, ARDS, ICU, Model, Scenario, SARS-CoV-2

## Abstract

**Hintergrund:**

Auch im Herbst 2021 droht trotz der steigenden Impfquoten gegen SARS-CoV-2 aufgrund saisonaler Veränderungen und der damit verbundenen erhöhten Infektionsraten eine erneute Infektionswelle mit einhergehender erneuter möglicher starker Belastung der Intensivmedizin. Im Folgenden sind verschiedene Szenarien mithilfe mathematischer Modelle simuliert, die unter der Annahme bestimmter Voraussetzungen eine Einschätzung hinsichtlich der Auslastung der Intensivbettenkapazitäten im Herbst in bestimmten Grenzen ermöglichen.

**Methodik:**

Die Simulation der Szenarien verwendet ein stationäres Modell, ergänzt um den Effekt der Impfungen. Das altersgruppenspezifische Risikoprofil für einen intensivpflichtigen Krankheitsverlauf wird anhand von Einweisungsdaten der 3. Welle auf Intensivstationen in Sentinel-Kliniken, den lokalen DIVI-Register-Belegungsdaten sowie den entsprechenden lokalen Inzidenzen durch lineare Regression mit Zeitversatz berechnet. Wir simulieren hierbei Impfquoten von 15 % für die U18-Kohorte, 70 % für die 15- bis 34-jährige Kohorte, 75/80/85 % für die Kohorte von 35 bis 59 Jahren sowie 85/90/95 % für die Ü60-Kohorte. Die Simulationen berücksichtigen, dass eine Impfung zu 100 % vor einem intensivpflichtigen Krankheitsverlauf schützt. Für den Schutz vor Infektion der Geimpften wurden die Simulationen zum einen für das Szenario Impfschutz für 70 % der Geimpften und zum anderen für das Szenario Impfschutz für 85 % der Geimpften dargestellt.

**Ergebnisse:**

Die Intensivbettenauslastung verläuft proportional zur Inzidenz. Der Faktor für dieses Verhältnis (Proportionalitätsfaktor) ist höher als in der 2. und 3. Welle, sodass vergleichbare Intensivbettenbelegungen erst bei höherer Inzidenz erreicht werden. Eine 10 %ige Steigerung der Impfquoten der Ü35 auf 85 % und der Ü60 auf 95 % führt zu einer erheblich verringerten Intensivbettenbelegung.

**Diskussion:**

Es besteht auch in den kommenden Monaten eine enge und lineare Beziehung zwischen der SARS-CoV-2-Inzidenz und der Intensivbettenbelegung. Bereits ab Inzidenzen von 200/100.000 ist wieder eine erhebliche Belastung der Intensivstationen mit mehr als 3000 COVID-19-Patienten zu erwarten, sofern die Impfquote nicht noch deutlich gesteigert wird. Wenige Prozentpunkte in der Impfquote haben eine erhebliche Auswirkung auf die potenzielle Intensivbelegung im Herbst, sodass Bemühungen um die Steigerung der Impfakzeptanz in den kommenden Wochen im Vordergrund stehen sollten. Für die Intensivmedizin ist die Impfquote der über 35-Jährigen von entscheidender Bedeutung.

**Zusatzmaterial online:**

Die Online-Version dieses Beitrags (10.1007/s00063-021-00862-9) enthält 3 weitere Abbildungen mit ergänzenden Daten zur Prognose der Intensivbettenbelegung, falls der Impfschutz gegenüber der COVID-19-Infektion auf 70 % reduziert ist.

## Abstract (en)

**Background:**

Despite the increasing vaccination rates against SARS-CoV‑2, there is a risk of a renewed wave of infections in autumn 2021 due to the high seasonality of the pathogen, with the associated renewed possible heavy burden on intensive care. In the following manuscript we simulated different scenarios using defined mathematical models to estimate the burden of intensive care treatment by COVID-19 patients within certain limits during the coming autumn.

**Methods:**

The simulation of the scenarios uses a stationary model supplemented by the effect of vaccinations. The age group-specific risk profile for intensive care unit (ICU)-associated disease progression is calculated using third wave ICU admission data from sentinel hospitals, local DIVI registry occupancy data and the corresponding local incidence rates by linear regression with time lag. We simulated vaccination rates of 15% for the over 18-year-old cohort, 70% for the 15–34 year cohort, 75%/80%/85% for the 35–59 year cohort and 85%/90%/95% for the over 60-year-old cohort. The simulations take into account that vaccination provides 100% protection against disease progression requiring intensive care. Regarding protection against infection in vaccinated persons the simulations are depicted for the scenario of 70% protection against infection in vaccinated persons and for the scenario of 85% protection against infection in vaccinated persons.

**Results:**

The incidence is proportional to ICU bed occupancy. The proportionality factor is higher than in the second and third waves, so that comparable ICU bed occupancy is only achieved at a higher incidence. A 10% increase in vaccination rates of the over 35-year-olds to 85% and of the over 60-year-olds to 95% leads to a significant reduction in ICU bed occupancy.

**Discussion:**

There will continue to be a close and linear relationship between SARS-CoV‑2 incidence and ICU bed occupancy in the coming months. Even above incidences of 200/100,000 a considerable burden of ICUs with more than 3000 COVID-19 patients can be expected again, unless the vaccination rate is significantly increased. A few percentage points in the vaccination rate have a significant impact on potential ICU occupancy in the autumn, so efforts to increase vaccination acceptance should be a priority in the coming weeks. For intensive care medicine, the vaccination rate of those over 35 years of age is crucial.

## Einleitung

Im Laufe der COVID-19-Pandemie sind in den vergangenen Monaten in Deutschland zwischenzeitlich bis zu 6000 Patienten täglich gleichzeitig intensivmedizinisch behandelt worden (www.intensivregister.de), mit einer hohen Sterblichkeit von etwa 50 % aller Beatmungsfälle [7, 9, 10]. Nachdem uns die Pandemie sowohl intensivmedizinisch als auch gesamtgesellschaftlich seit Anfang 2020 permanent in Atem hält [11] und es 2020 nur die Möglichkeit der nichtpharmazeutischen Interventionen zum Schutz vor dem Virus gab [5], steht seit Anfang 2021 eine sehr gut wirksame Impfung zur Verfügung [2, 6, 8], die sukzessiv im ersten Halbjahr 2021 zu einer Reduktion der Belastung des Gesundheitssystems geführt und mit dazu beigetragen hat, dass die dritte Welle abgeebbt ist.

Aus infektionsepidemiologischer Sicht war das Jahr 2020 geprägt vom Wildtyp des Virus, gefolgt von der Alpha-Variante im Frühjahr 2021, die sich als dritte Welle sehr schnell durchgesetzt hat [4]. Mit der weiteren globalen Verteilung des Virus ist es zunehmend zum Auftreten der noch infektiöseren Delta-Variante gekommen, die sich mittlerweile in ganz Europa durchgesetzt hat [1, 3]. Hier zeigen die Zahlen z. B. aus Großbritannien, wo die Delta-Variante schon früher aufgetreten und dominant geworden ist, bereits im Frühsommer erneut ansteigende Infektionszahlen und eine erneut zunehmende Belegung der Intensivstationen mit an COVID-19 erkrankten Intensivpatienten (NHS Situation Report; [13]).

Das Ziel der vorliegenden Arbeit ist es daher, unter Berücksichtigung der erhöhten Infektiosität der Delta-Variante [3] und des zeitgleich wachsenden Impfschutzes der Bevölkerung die Intensivbettenbelegung im Herbst/Winter 2021 als eine der Größen, die eine Belastung des Gesundheitssystems anzeigt, zu simulieren. Eine Prognose ist aufgrund der nicht verlässlich vorhersehbaren Entwicklung der Inzidenzen (Impfquoten und Umsetzung nichtpharmakologischer Maßnahmen) zum jetzigen Zeitpunkt nicht möglich. Es können jedoch Szenarien simuliert werden, die im Herbst/Winter 2021 realistisch eintreten könnten und anhand derer die Auslastung der intensivmedizinischen Kapazitäten eingeschätzt werden kann.

## Methode und Daten

Zur Simulation der Intensivbettenbelastung bei verschiedenen Impfszenarien in Abhängigkeit von altersstratifizierten Inzidenzen verwenden wir den im DIVI-Prognosetool (www.divi.de) implementierten und in einer vorangegangenen Publikation im Detail beschriebenen Ansatz [12]. Die Altersstratifizierung der Bevölkerung wird anhand der vom RKI-Dashboard vorgenommenen Alterseinteilung in den folgenden Altersgruppen durchgeführt: AG1: 0–4 Jahre, AG2: 5–14 Jahre, AG3: 15–34 Jahre, AG4: 35–59 Jahre, AG5: 60–79 Jahre, AG6: 80+ Jahre.

Das altersgruppenspezifische Risikoprofil für einen intensivpflichtigen Krankheitsverlauf wird anhand von Einweisungsdaten auf Intensivstationen in Sentinel-Kliniken, den lokalen DIVI-Belegungsdaten sowie den entsprechenden lokalen Inzidenzen durch lineare Regression mit Zeitversatz [12] berechnet. Aus den Sentinel-Kliniken Land Berlin, Klinikum Köln-Merheim sowie Universitätsklinikum Aachen lagen für die vorliegende Studie das Alter, Dauer der Hospitalisierung, Datum der Einweisung sowie, soweit erfolgt, die Dauer der intensivmedizinischen Behandlung vor. Aus den insgesamt über 5000 Datensätzen wird die altersgruppenspezifische Verteilung der Aufenthaltsdauer auf Intensivstation im zeitlichen Verlauf der Pandemie berechnet. Da die Datenlage für Intensivpatienten mit Delta-Mutante für die Analyse nicht ausreicht, werden die Aufenthaltsverteilung sowie das Risikoprofil r_k_ für die Altersgruppen k = 1 … 6 für die Alpha-Mutante für die Simulationen verwendet (Online-Supplement). Da die Delta-Mutante tendenziell als virulenter als die Alpha-Mutante eingestuft wird, wird der hierdurch induzierte Bias in den Modellsimulationen die Intensivbettenbelastung tendenziell eher unterschätzen.

Der Einfluss der Impfungen sowie der (als gleichwertig angenommenen) Immunisierung durch Genesung von einer vorhergegangenen Infektion mit COVID-19 wird durch ein Splitting der Population in den Altersgruppen AG_k_, k = 1 … 6 in jeweils eine immunisierte und eine nichtimmunisierte Altersgruppe vorgenommen: AG_k_ = [AG_k,i_, AG_k,ni_]. Quantitativ wird das Splitting mithilfe der Berechnung der bisher infizierten Patienten der jeweiligen Altersgruppe unter Berücksichtigung einer Dunkelziffer von 100 % angenommen. Daraus ergibt sich für die nichtimmunisierte Altersgruppe k:

1 $$AG_{k,ni}=AG_{k,\mathrm{pre}-\text{pandemic}}-2\sum _{j}I_{k,j}-V_{k}$$

wobei I_k,j_ die Zahl der Neuinfektionen in der Altersgruppe k am Tag j (Quelle: RKI-Dashboard) und V_k_ die Zahl der Doppelimpfungen in der Altersgruppe k (geschätzt aus RKI-Impfreport sowie simulierten Impfszenarien) darstellt. Da das Ziel der vorliegenden Studie eine Simulation des Intensivbettenbedarfs im Herbst 2021 ist, gehen wir von einer vollständigen (2-fachen oder äquivalenten) Impfung der impfwilligen Kohorte aus. Wir simulieren hierbei Impfquoten von 15 % für die U18-Kohorte, 70 % für die 15- bis 34-jährige Kohorte, 75/80/85 % für die Kohorte von 35 bis 59 Jahren sowie 85/90/95 % für die Ü60-Kohorte in allen Kombinationen. Die Simulationen berücksichtigen, dass eine Impfung für einen Anteil λ der Geimpften keinen Schutz vor Infektion bietet (simuliert werden λ = 15 % oder λ = 30 % entsprechend einem Infektionsschutz von 85 % oder 70 %), jedoch zu 100 % vor einem intensivpflichtigen Krankheitsverlauf schützt. Des Weiteren berücksichtigen wir a) eine gleichmäßige Verteilung der Infektionen I_k_ in Altersgruppe k über die nichtimmunisierte Kohorte:

$$I_{k}\sim \left(AG_{k,ni}+\lambda AG_{k,i}\right)$$

und b) eine überproportionale Infektion der jüngeren Alterskohorten entsprechend der Infektionsdynamik Anfang Juli 2021. Verteilung a) entspricht dabei einer langfristig stabilen Verteilung, wohingegen die Verteilung b) in einer von „superspreading“ getriebenen Infektionsdynamik wie im Juni/Juli 2021 vorherrscht.

Die Intensivbettenbelegung ist dann für jede Altersgruppe k proportional zu den Infizierten I_k_, gewichtet mit dem altersspezifischen Risikofaktor r_k_, wobei jedoch berücksichtigt werden muss, dass nur der Anteil der nichtimmunisierten Infizierten berücksichtigt werden darf. Hieraus ergibt sich für die intensivpflichtigen Krankheitsverläufe bei gegebener Inzidenz I_k_:

2 $$I_{\mathrm{risk},k}=r_{k}I_{k}\frac{AG_{k,ni}}{AG_{k,ni}+\lambda AG_{k,i}}$$

Aus dem Risiko der intensivpflichtigen Erkrankungsverläufe I_risk,k_ erhalten wir dann wie in [12] beschrieben die Belegungen der intensivmedizinischen Kapazitäten durch eine Faltung von I_risk,k_ mit der Verteilung der altersspezifischen Aufenthaltsdauern.

Caveat: Nach Gl. 2 nimmt bei gleicher Inzidenz I_k_ die Zahl der intensivpflichtigen Verläufe ab, wenn der Infektionsschutz der Impfung abnimmt (dementsprechend $\frac{AG_{k,ni}}{AG_{k,ni}+\lambda AG_{k,i}}$ zunimmt). Dieses scheinbare Paradoxon entsteht dadurch, dass die Impfdurchbrüche zwar zu den Inzidenzen beitragen, nicht jedoch zu den intensivpflichtigen Verläufen, sodass bei gleicher Inzidenz mehr „unkritische“ Verläufe zu erwarten sind. Im Gegenzug sind bei gleichem Kontaktverhalten entsprechend mehr Infektionen zu erwarten, sodass bei gleichem Kontaktverhalten ein schwächerer Impfschutz umgekehrt zu deutlich mehr Infektionen führt, sodass insgesamt ein schwächerer Infektionsschutz sich nachteilig auswirkt.

## Ergebnisse

Wir simulieren Inzidenzen von 0 bis 750 Infektionen pro Woche und 100.000 Einwohner für die deutsche Population (83 Mio. Einwohner, Verteilung auf die Altersgruppen nach Bundesamt für Statistik) und analysieren im Detail die folgenden Kombinationen

- der Impfquoten vom 30.07.2021, eines pessimistischen Szenarios (5/18,4/70/75/85/85 %) und eines optimistischen Szenarios (5/18,4/70/85/95/95 %) für die sechs Altersgruppen
- mit einem Infektionsschutz von 85 und 70 %
- sowie einer gleichmäßigen Verteilung der Infektionen auf die nichtimmunisierten Kohorten und einer Infektionsdynamik mit höherer Gewichtung der jüngeren Kohorten am Beispiel der Daten von 23. Juli 2021.

Zur Berechnung des Effekts einer Steigerung der altersgruppenspezifischen Impfquote simulieren wir darüber hinaus alle Impfquotenkombinationen in der Altersgruppe 35–59 von 75 %/80 %/85 % und in der Altersgruppe Ü60 von 85 %/90 %/95 % mit den übrigen oben aufgeführten Parametrisierungen.

Zum Vergleich mit dem Verhalten in der 2. und 3. Welle simulieren wir die Intensivbettenbelegung zusätzlich noch jeweils ohne Impfung.

Das Modell berücksichtigt eine aktuell stabile Infektionsdynamik ohne ein exponentiell ansteigendes Infektionsgeschehen. Daher wird der R‑Wert in den zugrunde gelegten Kalkulationen nicht berücksichtigt.

**Figure 1:**
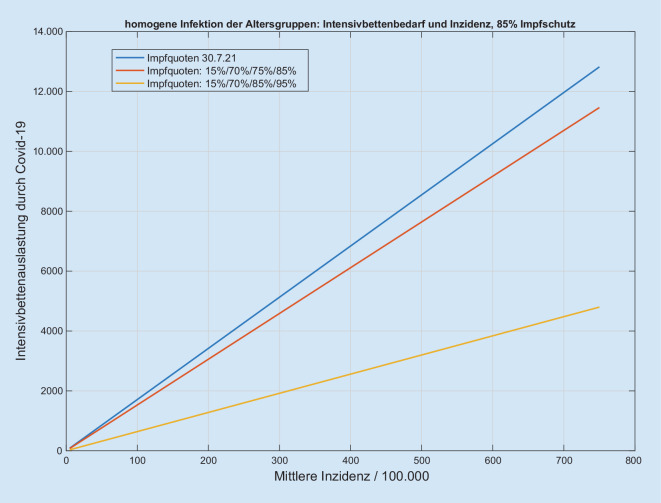

**Figure 2:**
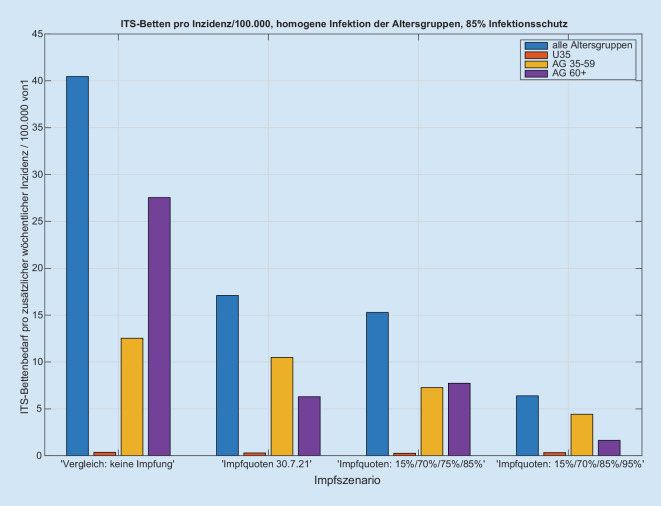

**Figure 3:**
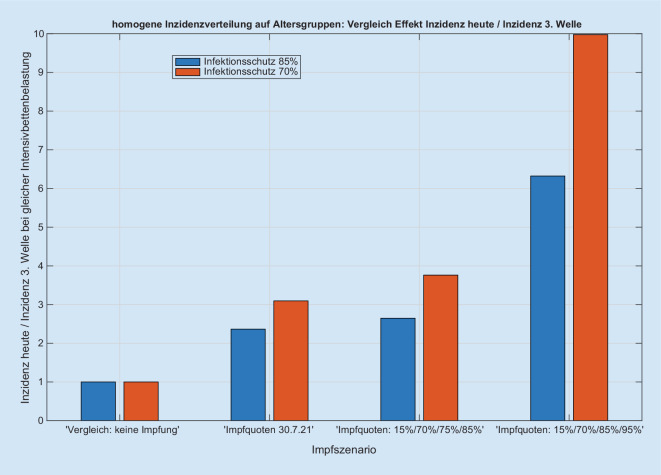

**Figure 4:**
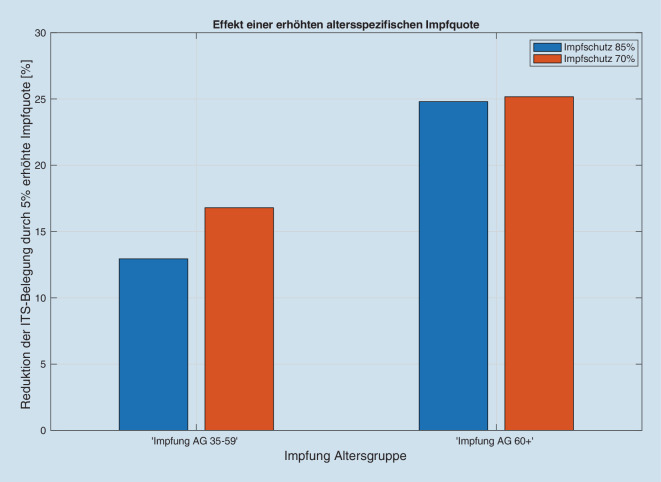

**Figure 5:**
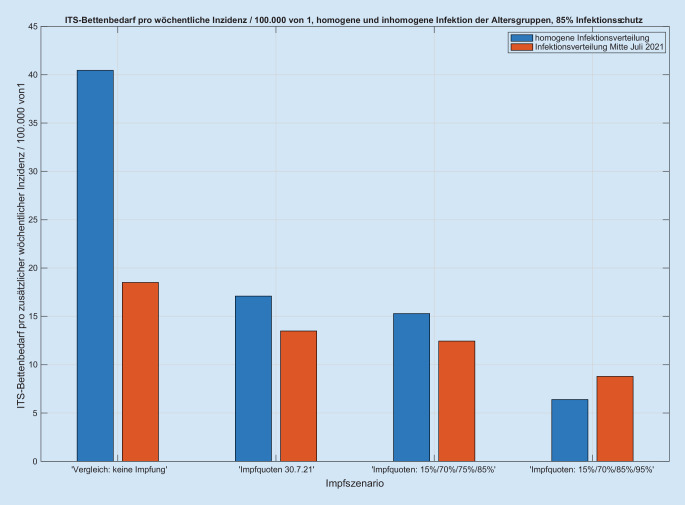

Wir erhalten für jede Kombination der o.g. Szenarien einen zu den Inzidenzen proportionalen Anstieg der Intensivbettenbelegungen (Abb. 1 und Abb. S1 im Online-Zusatzmaterial), wobei der Proportionalitätsfaktor vom jeweils simulierten Szenario abhängt (Abb. 2 und Abb. S2 im Online-Zusatzmaterial). Der Anteil der intensivpflichtigen Patienten an den Infizierten liegt zwischen 0,44 % (optimistisches Impfszenario) und 1,1 % (Impfstatus 30.07.2021) bei 85 % Impfschutz. Die Quotienten der Proportionalitätsfaktoren zwischen Intensivbettenbelegung und Inzidenz ergeben dann z. B. den Faktor zwischen den „gesellschaftlich und politisch tolerierten“ Inzidenzen, die heute zu einer vorgegebenen Bettenbelastung führen, und den respektiven Inzidenzen in Abhängigkeit von der Impfquote (Abb. 3). Den Einfluss einer Erhöhung der Impfquote auf die Intensivbettenbelegung (die mittlere Elastizität) in den Altersgruppen 35–59 sowie 60+ erhalten wir aus dem mittleren (gemittelt über alle anderen Parametervariationen) Verhältnis der jeweiligen Intensivbelastungen bei hoher/niedriger Impfquote (Abb. 4). Dies zeigt klar die hohe Bedeutung einer hohen Impfquote auch schon in der Altersgruppe 35–59, obwohl sie ein deutlich niedrigeres individuelles Risiko für einen schweren Erkrankungsverlauf im Vergleich zur Altersgruppe 60+ hat. Ein Vergleich der Simulationen für das Szenario einer homogenen Infektionsausbreitung auf die nichtimmunisierte Kohorte zu einer Infektionsdynamik primär innerhalb der jüngeren Altersgruppen zeigt wegen des deutlich niedrigeren individuellen Risikos r_k_ bei jüngeren Altersgruppen eine deutliche Reduktion der Intensivbettenbelegungen bei gleicher Gesamtinzidenz im letzteren Fall im Vergleich zu einer homogenen Infektion. In der Konsequenz folgt daraus umgekehrt, dass die Intensivbettenbelastung jedoch zunimmt, wenn die jüngeren Altersgruppen später die älteren Altersgruppen infizieren, obwohl die Gesamtinzidenz unverändert bleibt oder sogar abnehmen kann (Abb. 5 und Abb. S3 im Online-Zusatzmaterial).

## Diskussion

Die vorliegende Szenarienmodellierung zeigt einen Korridor der möglichen Intensivbettenbelastung mit an COVID-19 erkrankten Patienten im Herbst/Winter 2021 in Abhängigkeit vom Impffortschritt in der Bevölkerung. Besonders auffällig ist die weiterhin bestehende lineare Beziehung zwischen der Inzidenz der SARS-CoV-2-Infektion in der Bevölkerung und der möglichen Intensivbettenbelegung, auch wenn insbesondere die vulnerablen Patientengruppen geimpft sind (Abb. 1 und Abb. S1 im Online-Zusatzmaterial). Aus Sicht der Autoren ist es ist zudem offensichtlich, dass mit einer Impfquote von 75 % bei den über 35-Jährigen und 85 % bei den über 60-Jährigen bereits wieder ab einer Inzidenz von etwa 200/100.000 mehr als 3000 Intensivpatienten zeitgleich zu erwarten sind. Nach Meinung der Autoren ist durch den Impfschutz der vulnerablen Gruppen das Ausmaß steigender Infektionszahlen, wie sie während der ersten drei Wellen vorgelegen haben, im Hinblick auf ihre Bedeutung für die Auslastung der Intensivbettenkapazitäten mit COVID-19-Patienten im zweiten Halbjahr 2021 nicht mehr eins zu eins übertragbar. Vielmehr bedarf es im Vergleich zur dritten Welle im Hinblick auf die Intensivpflichtigkeit eines Korrekturfaktors, der je nach Impfquote etwa beim Faktor 2 bis 6 liegen wird (Abb. 3). Dies resultiert auch aus den unterschiedlichen Impfquoten der verschiedenen Altersgruppen und muss insbesondere dann angepasst werden, wenn viele Jüngere geimpft werden sollten.

Aus Sicht der Autoren zeigen die Simulationen auch, dass eine Steigerung der Impfquoten von 75 auf 85 % bei den über 35-Jährigen und von 85 auf 95 % bei den über 60-Jährigen zu einer erheblich geringeren Zahl potenzieller Intensivpatienten führen könnte (Abb. 2 und 4). Dieser kleine Unterschied von jeweils etwa 10 % wird aller Voraussicht nach auf die Intensivbettenbelastung mit an COVID-19 erkrankten Patienten einen erheblichen Einfluss haben. Es bleibt zudem zu bemerken, dass eine steigende Inzidenz verbunden ist mit einer prädominanten Steigerung der Intensivbelegung in der Altersgruppe 35–59 Jahre, aber kaum in der Gruppe unter 35 Jahren.

Die vorliegende Simulation der Auslastung der Intensivbetten mit an COVID-19 erkrankten Patienten zeigt, wie wichtig die Inzidenz als Frühwarnindikator bleibt und wie eng sie an eine schwere Erkrankung gekoppelt ist, auch wenn sie bezogen auf die Intensivbettenauslastung eines Korrekturfaktors bedarf. Trotz Simulation und Kalkulation der ITS-Betten-Kapazitäten sollte aus Sicht der Autoren nicht vergessen werden, dass eine Neuinfektion mit SARS-CoV‑2 für den betroffenen Patienten im Einzelfall zu einem schweren Krankheitsverlauf und im ungünstigsten Fall zum Tod des Patienten führen kann. Dies bedarf keines Korrekturfaktors! Hier bleibt es eine gesamtgesellschaftliche Verantwortung, drastisch steigende Inzidenzen zu vermeiden, insbesondere auch weil Kollateralschäden in der Intensivmedizin (Kapazitätsengpässe für intensivmedizinisch zu behandelnde Nicht-COVID-Patienten) und darüber hinaus im Langzeitverlauf für Patienten drohen. Auch mit der Delta-Variante erwarten die Autoren insbesondere vermehrt ungeimpfte über 35-jährige Patienten auf den Intensivstationen. Jüngere Patienten werden nach diesen Prognosen eher die Ausnahme bleiben. Der größte Unsicherheitsfaktor sind Impfdurchbrüche bei immunsupprimierten Patienten, die z. B. unter Medikamenten wie Rituximab oder bei Organtransplantierten zu erwarten sind. Auch ein nachlassender Impfschutz der hochaltrigen Bevölkerung könnte eine Rolle spielen. Zur Reduktion der möglichen Intensivbettenbelegung sollten diese Gruppen daher im Fokus eines maximalen Schutzes, z. B. durch eine dritte Impfung, nichtpharmazeutischer Interventionen oder der frühzeitigen Gabe neutralisierender Antikörper im Falle einer Infektion stehen.

### Warum erwarten wir bei einem 70 %igen Schutz durch die Impfung vor einer Infektion bei Geimpften weniger Intensivpatienten als bei einem 85 %igen Schutz durch die Impfung bei gleichen Inzidenzen?

Bei einem 70 %igen Impfschutz (Abb. S1 im Online-Zusatzmaterial) sind bei einer gegebenen Inzidenz 30 % der Geimpften zwar potenziell infektiös, aber sicher geschützt vor der Intensivaufnahme. Bei 85 %igem Impfschutz sind nur 15 % der Geimpften potenziell infektiös, aber vor Intensivaufnahme sicher geschützt. Bei einer gegebenen Inzidenz bedeutet dies, dass bei einem 85 %igen Impfschutz der Geimpften zum gegebenen Infektionsgeschehen/Inzidenz mehr Ungeimpfte beitragen. Gleichzeitig tragen die Ungeimpften aufgrund ihres höheren Risikos, schwer zu erkranken, zu einer Zunahme der Intensivbettenbelegung bei.

Eine Limitation der vorliegenden Arbeit ist die zum jetzigen Zeitpunkt nicht abschließend beurteilbare Wirksamkeit der Impfung bezogen auf den vollständigen Schutz vor der intensivpflichtigen Erkrankung im Rahmen der Infektion mit der Delta-Variante, insbesondere bei nachlassendem Impfschutz. Weiterhin können politische Entscheidungen und das Impfverhalten ebenso wenig vorhergesagt werden wie das Kontaktverhalten der Bevölkerung, sodass die vorliegende Arbeit einen Korridor vorgibt, indem sich der Herbst bewegen wird. Eine letzte Limitation ist eine mögliche, aber unwahrscheinliche Überschätzung der Höhe der Intensivbettenauslastung nach Inzidenzen bei reinen „superspreading events“ in ganz jungen Bevölkerungsgruppen, wie z. B. Konzerten mit vielen Tausend Ungeimpften und Ungeschützten in Innenräumen ohne Abstand. Dies halten die Autoren aber als isolierte „superspreading events“ im Herbst für sehr unwahrscheinlich, insbesondere auch weil nachfolgend in der Regel in einem solchen Fall auch Ältere infiziert werden.

Zusammenfassend besteht auch in den kommenden Monaten weiterhin eine lineare Beziehung zwischen der Inzidenz und der Intensivbettenauslastung mit an COVID-19 erkrankten Patienten, was nicht oft genug betont werden kann. Bereits ab Inzidenzen von 200/100.000 ist wieder eine erhebliche Belastung der Intensivstationen zu erwarten, sofern die Impfquote nicht noch deutlich gesteigert wird. Wenige Prozentpunkte in der Impfquote haben eine erhebliche Auswirkung auf die Intensivbelegung im Herbst, sodass Bemühungen um Steigerung der Impfquote und die Diskussion um die 3. Impfung in vulnerablen Gruppen in den kommenden Wochen im Vordergrund stehen sollten. An dieser Stelle möchten die Autoren darauf hinweisen, dass die Impfquoten in Deutschland auch bei den Älteren derzeit immer noch mit 80 % unzureichend sind und deutlich unterhalb der erreichten Impfquoten in anderen europäischen Ländern liegen.

## Supplementary Information




