# Supplementary material for: Intensive care bed requirements for COVID-19 in the fall/winter of 2021: Simulation of different scenarios under consideration of incidences and vaccination rates
Source: Med Klin Intensivmed Notfmed. 2021 Aug 9;117(6):439–46. [Article in German] doi: 10.1007/s00063-021-00862-9 (PMC8351237; doi:10.1007/s00063-021-00862-9)
Supplement: Supplementary file 1 [file 63_2021_862_MOESM1_ESM.pdf]

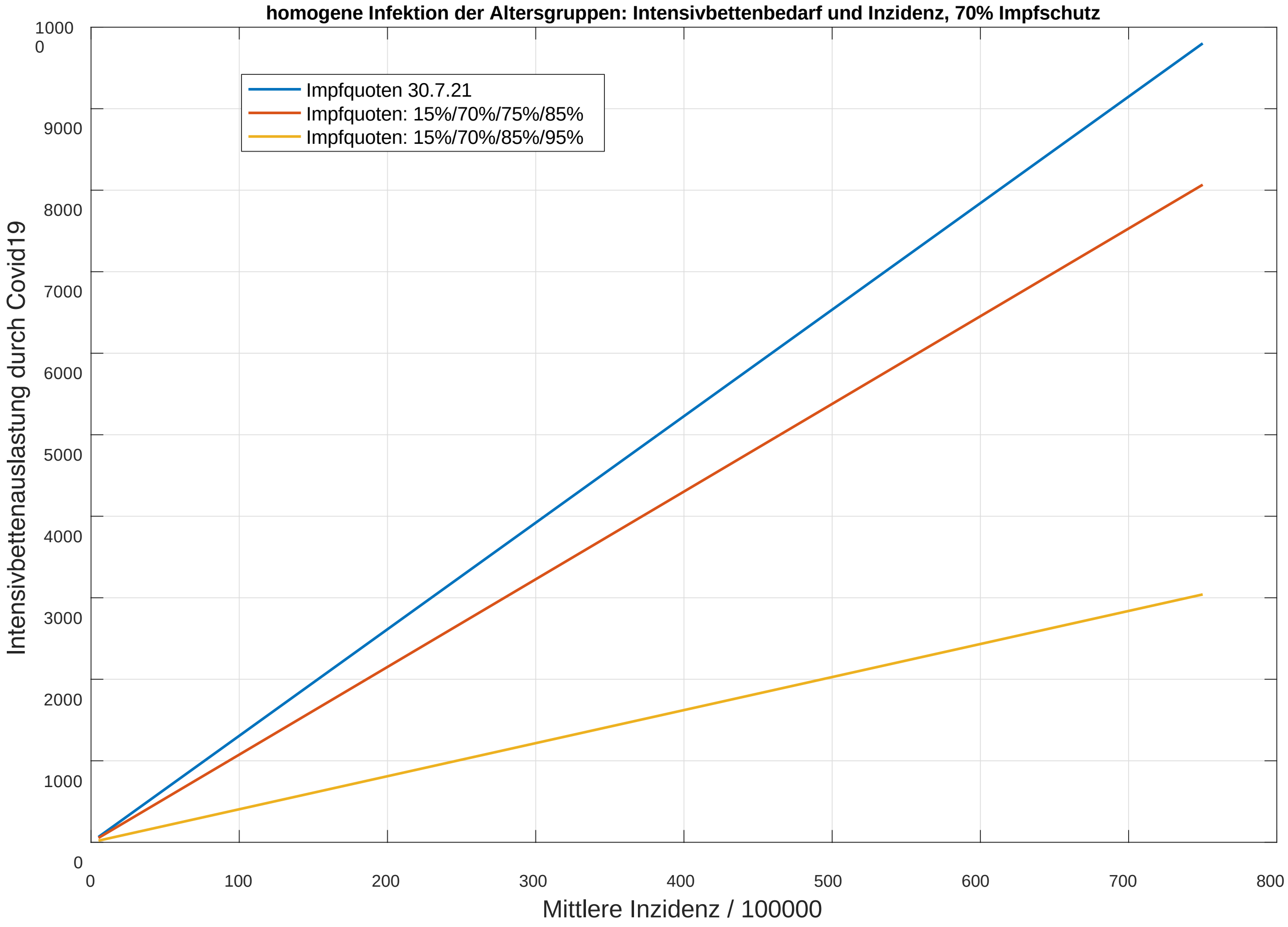

Abbildung S1: Intensiv-Bettenbedarf: 70% Impfschutz, a) Impfquoten 30.7., b) pessimistisches Szenario (U15: 15%, 15-34: 70%, 35-59: 75%, Ü60: 85%), c) optimistisches Szenario (U15: 15%, 15-34: 70%, 35-59: 85%, Ü60: 95%)

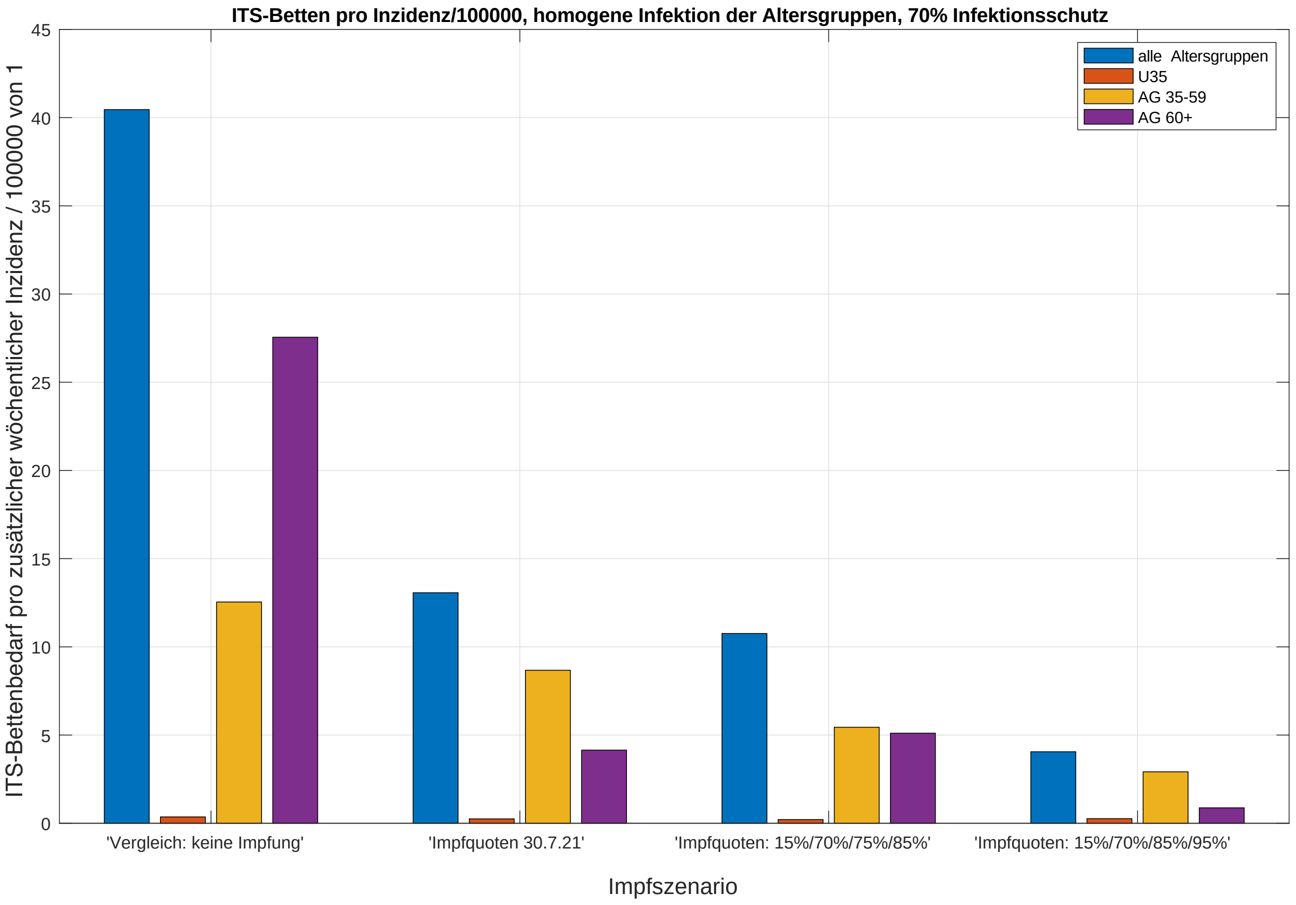

Abbildung S2: Zunahme des Intensiv-Bettenbedarfs pro Infektion / Woche auf 100000 Einwohner bei homogener Verteilung der Infektionen auf die Altersgruppen, Impfschutz 70%

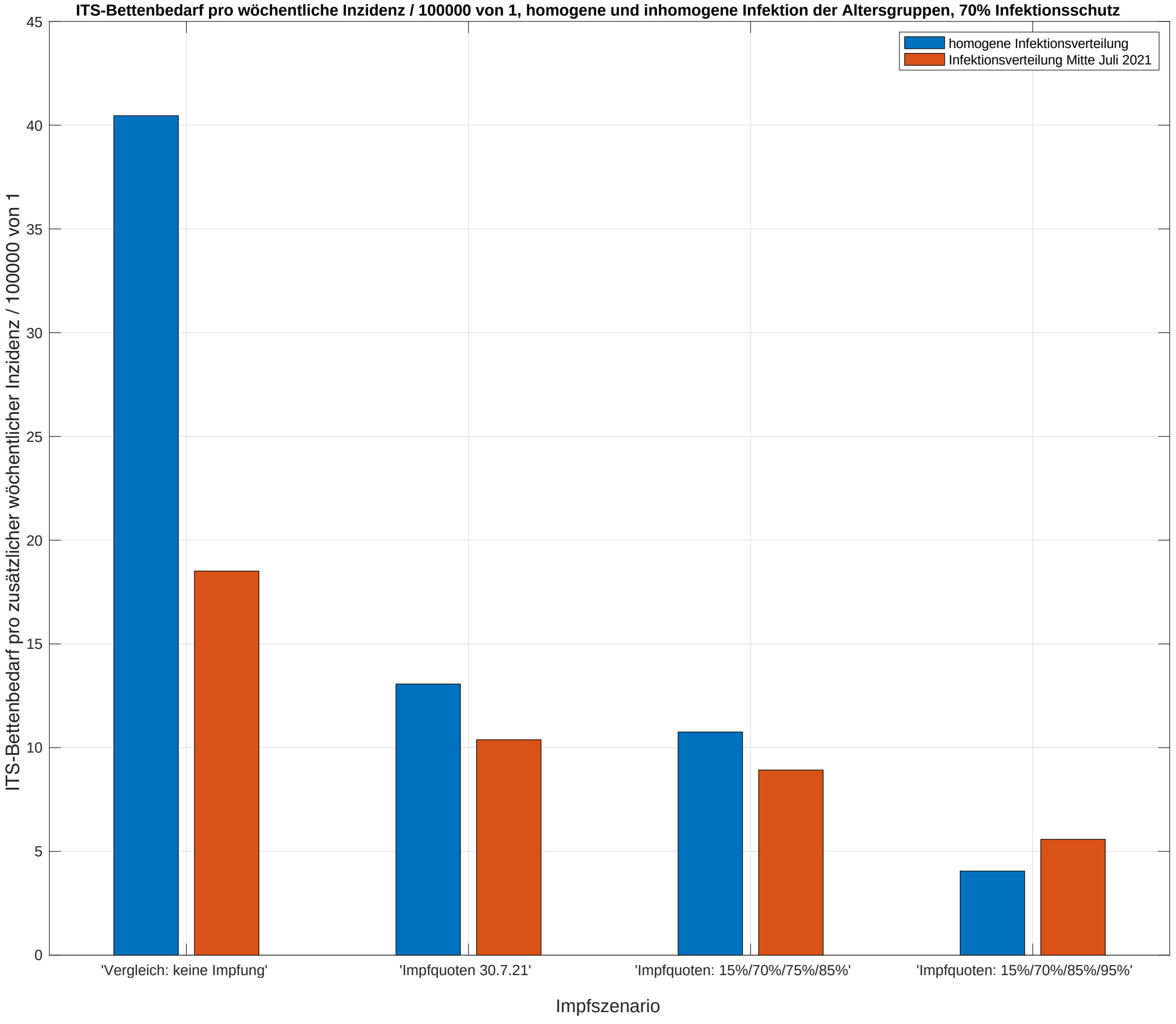

Abbildung S3: Impact einer auf jüngere Kohorten konzentrierten Infektionsdynamik auf den Intensiv-Bettenbedarf / (Wocheninzidenz/100000 Einwohner) – 70% Impfschutz
